# A purified, fermented, extract of *Triticum* aestivum has lymphomacidal activity mediated via natural killer cell activation

**DOI:** 10.1371/journal.pone.0190860

**Published:** 2018-01-05

**Authors:** Gustavo A. Barisone, Robert T. O’Donnell, Yunpeng Ma, Mastewal W. Abuhay, Kathleen Lundeberg, Sonia Gowda, Joseph M. Tuscano

**Affiliations:** 1 Division of Hematology and Oncology, Department of Internal Medicine, University of California Davis, Sacramento, California, United States of America; 2 Department of Veterans Affairs, Northern California Healthcare System, Mather, California, United States of America; Northwestren University, UNITED STATES

## Abstract

Non-Hodgkin lymphoma (NHL) affects over 400,000 people in the United States; its incidence increases with age. Treatment options are numerous and expanding, yet efficacy is often limited by toxicity, particularly in the elderly. Nearly 70% patients eventually die of the disease. Many patients explore less toxic alternative therapeutics proposed to boost anti-tumor immunity, despite a paucity of rigorous scientific data. Here we evaluate the lymphomacidal and immunomodulatory activities of a protein fraction isolated from fermented wheat germ. Fermented wheat germ extract was produced by fermenting wheat germ with *Saccharomyces cerevisiae*. A protein fraction was tested for lymphomacidal activity *in vitro* using NHL cell lines and *in vivo* using mouse xenografts. Mechanisms of action were explored *in vitro* by evaluating apoptosis and cell cycle and *in vivo* by immunophenotyping and measurement of NK cell activity. Potent lymphomacidal activity was observed in a panel of NHL cell lines and mice bearing NHL xenografts. This activity was not dependent on wheat germ agglutinin or benzoquinones. Fermented wheat germ proteins induced apoptosis in NHL cells, and augmented immune effector mechanisms, as measured by NK cell killing activity, degranulation and production of IFNγ. Fermented wheat germ extract can be easily produced and is efficacious in a human lymphoma xenograft model. The protein fraction is quantifiable and more potent, shows direct pro-apoptotic properties, and enhances immune-mediated tumor eradication. The results presented herein support the novel concept that proteins in fermented wheat germ have direct pro-apoptotic activity on lymphoma cells and augment host immune effector mechanisms.

## Introduction

Current therapeutic approaches for patients with non-Hodgkin lymphoma (NHL) include chemotherapy, signal transduction inhibitors, radiation and immunotherapy; bone marrow transplantation has become more frequent for patients who fail initial therapies. Although these treatments are often initially successful, most patients eventually become refractory and die of the disease. NHL is the sixth most common cause of cancer-related death in the United States [1–3]. The median age of lymphoma patients is 66 years old. The fastest growing segment of the population acquiring NHL is elderly males. Many of these patients cannot tolerate standard chemotherapy, hence efficacy is severely limited by toxicity. Therefore, less toxic, more effective therapeutics are needed.

According to a U.S. government survey, approximately 38% of adults and 12% of children use some form of complementary and alternative medicine (CAM) [4]. The use of many forms of CAM has significantly increased in prevalence over the last few decades. Although not specifically studied, the use of CAM in oncology patients is likely even higher due to a desperate attempt of cancer patients to find therapies that are perceived to be more effective and less toxic than conventional medicines. While the use of CAM has increased, scientific research has provided little evidence of their efficacy. There needs to be better, more rigorous scientific studies to back up these claims before they can be recommended for clinical use.

Growth inhibition of Ehrlich ascites tumor can be achieved by treatment of tumor-bearing mice with a mixture of 2, 6-dimethoxy-*p*-benzoquinone (DMBQ, present in wheat germ) and ascorbic acid (present in many plants) [5]. This mixture produces long-lived semiquinone and ascorbic free radicals. Quenching of quinone and ascorbic radicals depends on an NADPH-dependent, SH-containing enzymes [6]. The cytotoxicity of the free radical mixture is purported to be associated with the decreased NADP-reducing capacity of tumor cells [7]. During the fermentation of wheat germ, quinones are released by yeast glycosidases. Based on the hypothesis that quinones are “immunostimulatory”, Szent-Györgyi produced a dried extract of wheat germ fermented by *Saccharomyces cerevisiae* [8]. The extract has been standardized to DMBQ content and named MSC (trade name: AVEMAR) [9–11]. Crude fermented wheat germ extract (FWGE) has been shown to be cytotoxic in various malignant cells lines, including T-cell leukemia [12, 13], colorectal carcinoma [14, 15], promyelocytic leukemia [16], hepatocellular carcinoma [17], pancreatic carcinoma [18] and ovarian carcinoma [19, 20], as well as neuroblastoma, melanoma and testicular, cervical, thyroid and lung carcinoma-derived cell lines [20]. Anti-tumor and anti-metastatic activity of FWGE in animal models has been reported in melanoma and in squamous cell, lung and colorectal carcinomas [9, 10, 21–23]; preliminary clinical trial data in melanoma [24] and colorectal carcinoma [23, 25] are promising. FWGE has been reported to be immunostimulatory [11, 13, 14, 25, 26] and beneficial in systemic lupus erythematosus [27] and other autoimmune diseases [28, 29], and in the supportive care of cancer patients [30, 31]. FWGE consists of hundreds to thousands of molecules [32]; its active components, targets or mechanisms of action are largely unknown. It has been hypothesized that FWGE anti-tumor activity is based on its content of benzoquinones [32], fiber, lipids and phytic acid.

Based on our experience with a mantle cell lymphoma patient that derived objective benefit from self-administered FWGE, we created a semi-purified, reproducible formulation and further explored its therapeutic potential against NHL. Here we report *in vitro* and *in vivo* anti-tumor activity and mechanistic data of a protein extract of FWGE, called fermented wheat germ proteins (FWGP).

## Materials and methods

### Production and purification of FWGE and FWGP

Fresh wheat germ (Northern Edge, Randolph & James Flax Mills Ltd, Prince Albert, Saskatchewan, Canada) was dry-blended at 4°C to flour quality. Fifty grams of wheat germ powder were mixed with 16 g of dehydrated baker’s yeast in 500 ml distilled water and incubated at 28–30°C for 48 hours while shaking at 225–300 rpm in a 1000 ml flask. The supernatant was collected after centrifugation (9,500 x *g*, 4°C, 35 minutes) and either freeze-dried and labeled FWGE or subject to fractionation as follows. To produce FWGP, the post-fermentation supernatant was precipitated with ethanol (70% final concentration) overnight at -20°C and centrifuged (9,500 x *g*, 4°C, 35 minutes); the pellet was frozen at -80°C and lyophilized for 2–3 days until dry. Typically, 2 g of lyophilized powder were resuspended in 40 ml PBS and allowed to completely solubilize by stirring at 4°C for up to 24 hours. Any insoluble material was discarded; the preparation sterilized by filtration through 0.2 μm PES membranes (Millipore) and applied to a Sephadex G50 column. The eluent was assessed for lymphomacidal activity and the most potent fractions were combined, vacuum-dried, re-dissolved in PBS and applied to a Superdex S200 column. Elution fractions were collected, assessed for lymphomacidal activity and the most potent fractions were combined, vacuum-dried, and designated as FWGP. Protein content was quantified by BCA assays (Thermo Fisher). Aliquots were stored at -80°C until ready for use.

### Cell lines and primary specimens

Lymphoma (Ramos, Raji, DOHH-2, Granta-519, Sudhl4, Chevalier, WSU-WM, BM35, DG75), T-cell leukemia (Jurkat), lung (H1650), breast (MCF-7) and hepatic (HepG2) cancer cell lines were purchased from ATCC (Rockville, MD) and grown in RPMI-1640 or DMEM supplemented with 10% heat-inactivated fetal bovine serum (HI-FBS), 100 units/ml penicillin G, and 100 μg/ml streptomycin sulfate at 37°C in 5% CO_2_ and 90% humidity according to ATCC recommendations. Fresh vials of cells were periodically thawed and used for *in vitro* experiments to ensure that changes to cells have not occurred over time/passages in culture. For xenograft studies, a fresh vial of Raji cells was thawed 7–10 days before tumor cell implantation. YAC-1 cells were grown in RPMI medium supplemented with 10% HI-FBS. K562 cells were grown in Iscove’s modified Dulbecco’s medium supplemented with 10% HI-FBS.

Human peripheral blood mononuclear cells (PBMCs) from healthy donors were isolated from whole blood collected in citrated vacuum tubes using standard protocols. Blood was diluted 1:1 with PBS, layered over Ficoll-Paque Plus (GE Healthcare) and centrifuged for 30 minutes at 400 x *g*, 25°C. The buffy coat was collected, washed twice with PBS and the cells were resuspended in RPMI-1640 supplemented with 10% HI-FBS, 300 mg/L glutamine and penicillin/streptomycin. Untouched natural killer (NK) cells were isolated from fresh PBMCs using a magnetic purification system (Miltenyi Biotech). Briefly, 10^8^ PBMCs in 400 μl buffer were incubated with 100 μl biotin-antibody cocktail (5 minutes, 4°C) and 200 μl NK cell microbead cocktail (10 minutes, 4°C); the cell suspension was loaded in an LS column attached to a magnet and the NK-enriched unlabeled cells collected as the flow-through.

### Direct cytotoxicity

Direct cytotoxic activity of FWGE was assayed by incubating 5 x 10^4^ cells/well (96-well plates) in 100 μl culture medium with the indicated concentrations of FWGE for up to 72 hours at 37°C, 5% CO_2_. Cell viability was assessed using an MTS-based assay (Promega) according to the manufacturer’s instructions and compared to untreated controls. IC_50_ values were calculated by fitting the dose-response data to a dose–inhibition curve using GraphPad Prism software. Cytotoxicity of heat-inactivated FWGE (80°C, 90 minutes), proteinase K-treated FWGE (100 μg/ml, 37°C, 1 hour) and the protein fraction FWGP were assayed in the same way. Three replicate wells per condition were used in 3 independent experiments.

### Apoptosis and cell cycle

Raji or Ramos cells (1 x 10^6^/ml) were incubated with 200 μg/ml FWGP for 1, 3, 6, 12, 24 and 48 hours, washed with PBS and resuspended in 100 μl Annexin-V binding buffer (10 mM HEPES, 140 mM NaCl, 2.5 mM CaCl_2_, pH = 7.4) with 5 μl Annexin-V-Cy5 (BD Pharmingen) and 1 μg/ml Sytox Green (Thermo Fisher) according to the manufacturer’s instructions. After staining for 15 minutes, cells were analyzed by flow cytometry using a FACSCanto instrument (BD); 30,000 events per sample were acquired. Untreated cells were stained as above and used as controls. Untreated, unstained or single-stained controls were used for compensation. To assess caspase activity, cells were incubated with FWGP or PBS control as above and stained with a Vybrant FAM Poly Caspases Assay Kit (Molecular Probes) according to the manufacturer’s instructions. Briefly, 300 μl of cell suspension (1 x 10^6^ cells/ml) were incubated with VAD-FMK FLICA reagent and Hoechst 33342 for the detection of activated caspases 1, 2, 4, 5, 6, 8 and 9, washed and analyzed by flow cytometry as above. Data were analyzed using FlowJo software. For cell cycle analysis, cells were fixed in ethanol, washed, and stained with 20 μg/ml propidium iodide (PI) as previously described [33]; data (50,000 events/sample) were acquired as noted above.

### Cell staining and flow cytometry

Lymphocyte surface and activation markers were stained by resuspending 10^6^ cells in 10 μl Fc receptor block (TruStain fcX mouse or human, BioLegend) for 10 minutes at 25°C followed by 30 μl antibody cocktail for 30 minutes at 4°C in 96-well round-bottom plates. Cells were washed twice with PBS and stained with the fixable viability dye (FVD) Zombie near infra-red (ZNIR, BioLegend, 100 μl/well of a 1:1,000 dilution in PBS) for 15 minutes at room temperature. After washing with 2% FBS/PBS, samples were either acquired immediately or fixed in 4% paraformaldehyde/PBS for 10 minutes at 25°C. For intracellular staining, fixed cells were washed 3 times with permeabilization buffer (BioLegend) and incubated with the appropriate antibodies for 30 minutes at 25°C followed by 2 final washes and resuspension in 2% FBS/PBS. Data were acquired in an LRSFortessa (BD Biosciences, San Jose, CA) instrument equipped with an automated sampling module. All flow cytometry data were analyzed with FlowJo (Tree Star, Ashland, OR). Mouse-specific antibodies were as follows (clones indicated in between parenthesis): AF-700 anti-CD19 (6D5), PE/Cy5 anti-Ly6G/6C (RB6-8C5), PerCP/Cy5.5 anti-CD4 (RM4-5), BV570 anti-CD11b (M1/70), BV650 anti-CD25 (PC61), Pacific Blue anti-CD45 (30-F11), APC anti CD69 (H1.2F3), BV785 anti-CD3 (17A2), BV711 anti-CD8a (53–6.7), PE anti-CD49b (DX5), FITC anti-CD8a (53–6.7), PE/Cy7 anti-CD49b (DX5), AF647 anti-CD49b (DX5) and BV510 anti-CD3 (17A2) from BioLegend, PE anti-Granzyme B (NGZB) from eBioscience, and V450 anti-CD107a (1D4B) and AF-488 anti-IFNγ (XMG1.2) from BD Biosciences. Human-specific antibodies were PerCP/Cy5.5 anti-CD3 (OKT3), BV785 anti-CD56 (5.1H11), PE anti-CD69 (FN50), APC anti-CD25 (M-A251) from BioLegend, and AF-488 anti-INFγ (B27) from BD Biosciences.

### qPCR arrays

Quantitative real-time PCR (qPCR) was performed using the Apoptosis and Survival Tier 1–4 H384 panel (Bio-Rad PrimePCR) to examine over 350 genes associated with cell survival and apoptosis. Total RNA was extracted from control and treated (200 ng/μl) Raji cells at the indicated time points using an RNeasy kit (Qiagen) and reverse-transcribed with the iScript™ Advanced cDNA Synthesis Kit (Bio-Rad) according to the manufacturer’s instruction. Reactions were run in a 7900HT instrument (Applied Biosystems) using SsoAdvanced™ Universal SYBR^®^ Green Supermix (Bio-Rad). Data were normalized and analyzed with the PrimePCR analysis software (Bio-Rad). Selected genes were validated by immunoblotting.

### Immunoblotting

Five million Raji cells were incubated with 200 μg/ml FWGP or PBS control in 5 ml culture medium at 37°C, 5% CO_2_. At 2, 6, 12, 24 and 48 hours, 1-ml aliquots were collected, centrifuged and cells washed with PBS. Cell pellets were lysed in 100 μL of RIPA buffer (150 mM NaCl, 1% sodium deoxycholate, 0.1% SDS, 1% Triton X-100, 50 mM Tris-HCl, pH = 7.2) supplemented with protease inhibitors on ice for 30 minutes with occasional vortexing. Immunoblotting was done as previously described [34, 35]. Briefly, cell lysates (50 μg protein/lane in reducing Laemmli buffer) were run on a 10% SDS-PAGE gel and transferred to nitrocellulose. Membranes were blocked with 5% BSA or 5% non-fat dry milk in PBS and incubated with primary antibodies (4°C, overnight) diluted as indicated in 5% BSA in PBS with 0.01% Tween-20 (PBS-T). Membranes were washed with PBS-T and incubated for 1 hour at room temperature with HRP-labeled secondary antibodies, washed and developed with Luminata Crescendo (Millipore) detection reagent. Signal intensity was quantified using ImageJ software and normalized to load controls (GAPDH).

### Killing assays

Killing assays were performed by incubating effector and target cells at the specified ratios for 4 or 24 hours, followed by flow cytometric quantification of double-labeled target cells. For mouse samples, 0.5 μl CFSE (stock = 10 mM in DMSO, eBioscience) were added to 5 x 10^5^ target YAC-1 cells in 1 ml 5% HI-FBS/PBS in a 15-ml conical tube, mixed immediately and incubated for 5 minutes at room temperature. Labeling was stopped by adding 2–3 ml HI-FBS and culture medium to fill the tube. Cells were centrifuged (5 minutes, 300 x *g*, 24°C), resuspended at 1 x 10^6^ cells/ml in culture medium and allowed to recover overnight. Mouse splenocytes were T-cell depleted by incubating with 1.5 μg/10^6^ cells anti-Thy1.2 (BioLegend, clone 30-H12) for 30 minutes at 4°C, washing and incubating with rabbit serum complement (Cedarlane, Burlington, NC) at the lot-specific recommended dilution for 45 minutes at 37°C. T-cell depleted (TCD) splenocytes were then washed twice and resuspended in culture medium. Twenty thousand CFSE-YAC-1 cells were incubated with TCD splenocytes in 96-well round-bottom plates, in a final volume of 200 μl containing recombinant human IL-2 (rhIL-2, 1,000 IU/ml, Biological Resources Branch, NCI, Frederick, MD). After 4 h, cells were centrifuged, washed with PBS and resuspended in 100 μl FVD eFluor 455UV (1:1000 dilution in PBS, Thermo Fisher) for 30 minutes at 4°C. Cells were washed with 2% FBS/PBS and resuspended in the same buffer for acquisition in a Fortessa (BD) flow cytometer. Dead target cells were defined as the CFSE^+^FVD^+^ population.

Killing activity of human PBMCs was assayed by incubating PBMCs with the indicated concentrations of FWGP for 20 h at 37°C, 5% CO_2_. Cells were then washed twice with culture medium and counted; 2.5 x 10^4^ viable PBMCs/100 μl/well were incubated with an equal number of Ramos (target) cells for 24 h. Cytotoxicity was assessed using the DELFIA EuTDA-based assay (Perkin Elmer) and normalized to controls (target cells incubated with untreated PBMCs).

### Degranulation assays

TCD splenocytes (10^5^ cells/200 μl/well) were plated with YAC-1 cells at the indicated ratios in RPMI-1640, 10% HI-FBS, 1,000 IU/ml rhIL-2. V450 anti-CD107a (BD, clone 1D4B, 0.25 μg/well) was added and incubated at 37°C for 1h. Protein transport inhibitor cocktail (ThermoFisher) was then added to each well and further incubated for 4 h. Cells were then washed twice with PBS/2% FBS and stained for CD3 and CD49b. Dead cells were stained with ZNIR (BioLegend). Controls included effector-only cells (non-stimulated) and cells stimulated with PMA+ionomycin (Cell Stimulation Cocktail, Thermo Fisher).

### Animals

For xenograft experiments, female 6-8-week-old nu/nu mice (Harlan, Indianapolis, IN) were maintained in micro-isolation cages under pathogen-free conditions at the UC Davis animal facility. All procedures were conducted under an approved protocol according to national and institutional guidelines. Three days after whole body irradiation (400 rads), Raji human lymphoma cells (1 x 10^6^ in 100 μl PBS) were implanted subcutaneously on the left flank. Either on the day of tumor implantation (preemptive), or once approximately 300 mm^3^ tumors had been established (~20 days), mice were randomly divided into treatment groups (n = 8–10). Treatment (FWGE, FWGP or PBS) was administered by gavage once daily 5 days per week for the duration of the study. Tumors were measured twice per week using a digital caliper; tumor volumes were calculated using the equation: (length x width x depth) x 0.52. Tumor responses were categorized as follows: cure (C, tumor disappeared and did not re-grow by the end of the 84-day study); complete regression (CR, tumor disappeared for at least 7 days but later re-grew); partial regression (PR, tumor volume decreased by 50% or more for at least 7 days then re-grew).

Mice were euthanized when the tumor reached 15 mm in any dimension, if they showed signs of distress, or at the end of the 84-day study. Toxicity was assessed by twice-weekly measurement of weight, activity, and blood counts for the first 28 days, then weekly for the rest of the 84-day study period. Standard assessment of toxicity was performed by the UC Davis School of Veterinary Medicine Laboratory Animal Clinic.

For xenograft experiments with NK cell-depleted animals, female 6-8-week-old nu/nu mice were implanted with Raji cells as above and treatment with FWGP started once tumors had been established (defined as day 0). Anti-asialo-GM1 (Wako, Richmond, VA) was administered as 25 μl (according to lot-specific titration by the manufacturer) intraperitoneal injections on days 0, 10, 20 and 30.

For R-CHOP treatment, cyclophosphamide (in saline) was administered intraperitoneally (30 mg/kg; i.p.); doxorubicin (2.475 mg/kg) and vincristine (0.375 mg/kg), both in saline, were administered by bolus tail vein injections (i.v.); prednisone (in saline) was administered orally (0.15 mg/kg, p.o.), starting on day 1.

For studies of FWGP in immunocompetent animals, BALB/c 8-month-old female mice (Envigo) were treated with FWGP (140 mg/kg) by daily gavage for 3 days. On day 4, splenocytes were collected by dissecting spleens into 3 ml of cold RPMI and disrupting the tissue through 100-μm mesh. The suspension was further dissociated by passing sequentially through 20, 21 and 23 gauge needles, 3 times each. Red blood cells were lysed with ACK buffer (Thermo Fisher) for 5 minutes at room temperature, washed and resuspended in culture medium.

### Statistical analysis

*In vitro* cytotoxicity data were analyzed by a two-tailed, unpaired Student’s t-test. Experiments with 3 or more groups were analyzed using ANOVA or 2-way ANOVA with post-tests for multiple comparisons as indicated in each figure. For Kaplan-Meier curves, an “event” was defined as tumor volume reaching at least 1500 mm^3^. Each individual mouse was ranked as 1 (event occurred) or 0 (event did not occur) and the time to event (in days) was determined. When an individual was ranked as 0, a time to event of 88 days was recorded. Chi-squared and *p* values were determined by the Log-rank test. All statistical analysis was performed using GraphPad Prism software (San Diego, CA). Statistical significance is indicated as *p<0.05, **p<0.01, ***p<0.001 and ****p<0.0001.

### Ethics

All animal work has been conducted according to relevant national and international guidelines under approved protocols from the University of California Davis Institutional Animal Care and Use Committee (AAALAC accreditation #000029; PHS Animal Assurance #A3433-01; USDA Registration #93-R-0433). Human cells were collected from discarded leukapheresis bags under protocols approved by The University of California Davis Institutional Review Board Administration. Informed written consent was obtained at the time of collection. The need of consent for the of discarded, anonymized leukapheresis bags was waived by the ethics committee. No patient was recruited or sample collected for the sole purpose of this study.

## Results

### FWGE has potent *in vitro* lymphomacidal activity

**Fig 1:**
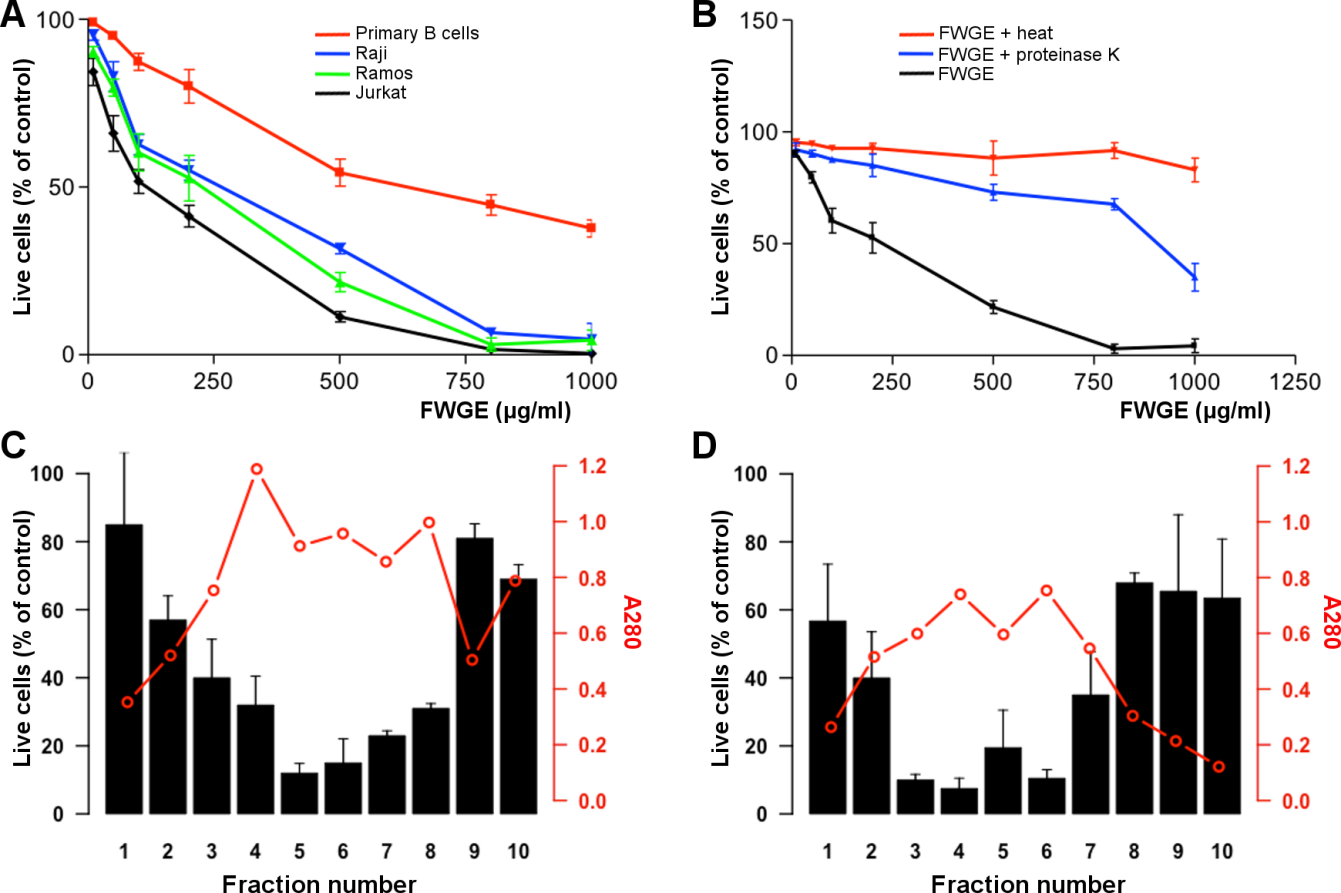
*In vitro* lymphomacidal activity of FWGE and FWGP. **(A)** Raji, Ramos, Jurkat and normal primary human B cells were incubated with increasing concentrations of fermented wheat germ extract (FWGE) for 72 hours. Viability was measured by MTS assay. Data points indicate the mean of 3 biological replicates, each in triplicate; error bars represent standard deviation. **(B)** FWGE was heated or treated with proteinase K and cytotoxicity was assessed as in (A). Cytotoxic activity against Raji cells (as % of trypan-blue negative cells relative to untreated controls) and protein chromatographic profile (as A280) of the ethanol-insoluble extract from FWGE separated on Sephadex G50 (**C**) and Superdex G200 (**D**).

The FWGE used in these studies was produced in-house by fermenting raw wheat germ with *Saccharomyces cerevisiae*. To assess if the *in vitro* activity of FWGE was equivalent to the commercially available product (AVEMAR^®^) *in vitro* cytotoxicity assays with both agents were done; the killing activity was equivalent (see S6 Fig). We initially assessed the cytotoxic activity of FWGE on two Burkitt lymphoma cells lines (Raji and Ramos) and the Jurkat T-cell leukemia cell line as compared to primary human B cells (**Fig 1A**). After 72 hours, FWGE showed considerable cytotoxic activity in the three cancer-derived cell lines with IC_50_ = 120, 250, and 275 μg/ml for Jurkat, Ramos and Raji, respectively. However, FWGE was substantially less cytotoxic to normal human primary B cells, as evidenced by an IC_50_ = 582 μg/ml, 2–5 times higher than that observed in malignant cells. Pretreatment of FWGE with proteinase K or heat completely abrogated its cytotoxic activity (**Fig 1B**), suggesting that the active component(s) of FWGE is a peptide. Moreover, the cytotoxicity of FWGE was dependent on a minimum of 8 hours of fermentation (data not shown).

### Direct cytotoxic activity of fermented wheat germ proteins

Soluble proteins in FWGE were ethanol-precipitated, dissolved in PBS, and passed through a Sephadex G50 column. The eluent fractions were assessed using SDS-PAGE and found to be between 10 and 200 kDa (not shown). When assessed for cytotoxicity using Raji cells, fractions 4–8 were the most potent; 50 μg/ml killed 70–90% of Raji cells (**Fig 1C**). These fractions were collected, vacuum-dried overnight, re-dissolved in PBS and further size-separated using Superdex S200. Eluted fractions were again assessed using SDS-PAGE and most were 10–100 kDa (not shown). All eluted fractions were assessed for cytotoxicity; fractions 3–6 killed 80–90% of Raji cells (**Fig 1D**). These fractions were combined for further analysis; they were now termed FWGP. FWGP was then assessed for cytotoxic activity and in a dose-response experiment using a panel of malignant cell NHL cell lines that represented a broad array and the most common B cell NHL subtypes; IC_50_ ranged from 20–150 μg/ml (**Table 1**). FWGP also showed cytotoxic activity against H1650 and A549 (lung carcinoma, IC_50_ = 144 and 70 μg/ml, respectively) and HepG2 cells (hepatic carcinoma, IC_50_ = 245 μg/ml), but very modest or no activity against MCF-7 cells (breast cancer, IC_50_ = 630 μg/ml). Comparison of IC_50_ of FWGE and FWGP in Raji and Ramos cells suggests that FWGP is significantly more potent in both cell lines (120 vs 39 and 250 vs 70 μg/ml, respectively). Since wheat germ agglutinin (WGA) is known to be cytotoxic [36] we sought to determine if WGA in these preparations was contributing to the lymphomacidal effect. WGA depletion by immunoprecipitation had no effect on the cytotoxic activity of FWGP (**S1 Fig**). WGA depletion was confirmed by immunoblot analysis (not shown).

**Table 1:** Cytotoxic activity of FWGP. FWGP was assessed for cytotoxic potential in a panel of human cancer cell lines. IC_50_s were calculated from dose-response (growth inhibition) curves.

Cell Line	Tumor Type	IC_50_ (μg/ml)
SUDHL4	NHL (diffuse large)	70
DG75	Lymphoblastoid	20
BM35	NHL (aggressive)	71
DoHH2	NHL (follicular)	171
Raji	NHL (Burkitt)	39
Ramos	NHL (Burkitt)	70
WSU-WM	NHL/Waldenstrom	42
Granta-519	NHL (mantle cell)	80
Chevalier	NHL	40
H1650	Lung	144
A549	Lung	70
MCF-7	Breast	639
HepG2	Hepatic	245

**Fig 2:**
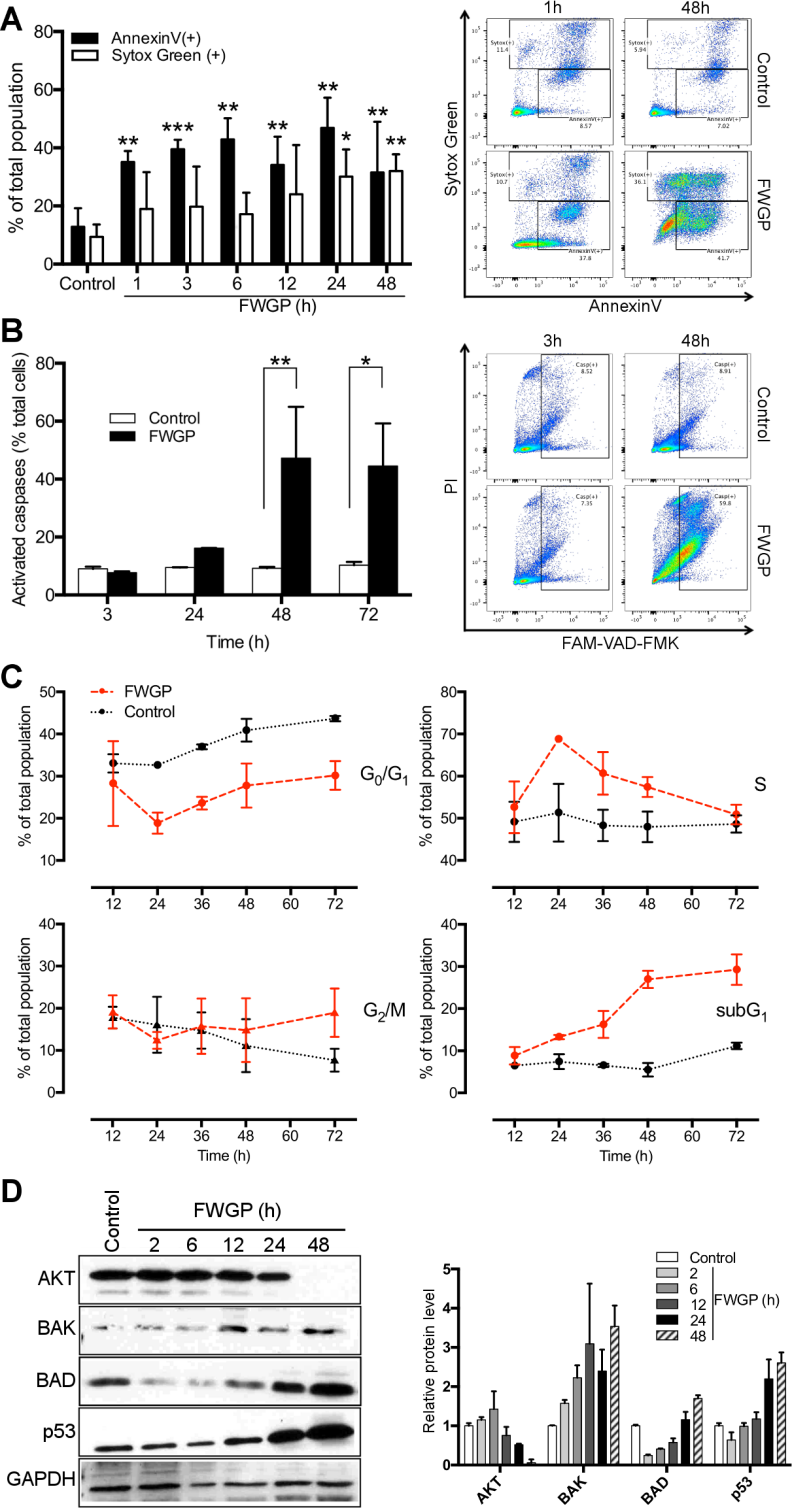
Effect of FWGP on apoptosis and cell cycle. **(A)** Early apoptosis was measured on Raji cells by flow cytometry after Annexin-V staining; Sytox Green was used for staining of late apoptotic/necrotic cells. Cells were incubated with FWGP (200 μg/ml) for the indicated times and compared to untreated controls. **(B)** Flow cytometric detection of activated caspases 1, 2, 4, 5, 6, 8 and 9 using FAM-VAD-FMK substrate; propidium iodide (PI) was used as a counterstain. Bars represent mean ± SD (n = 3, *p<0.05; **p<0.01; ***p<0.001). Representative flow cytometry plots on the right. **(C)** Cell cycle analysis of Raji cells treated with FWGP. DNA content was measured using PI staining of fixed cells. Cycle phase populations were calculated using a unidimensional model. Complete flow cytometry data are presented in S2–S4 Figs. **(D)** Immunoblot analysis of AKT, BAK, BAD and p53 in total cell extracts from Raji cells treated with FWGP (200 μg/ml) for the indicated times. Blot quantification is shown on the right as band intensity relative to the load control GAPDH.

To investigate the mechanisms by which FWGP exerts direct lymphomacidal activity, we assessed FWGP-treated Ramos cells for apoptosis by staining with Annexin V, with Sytox Green counterstaining to differentiate late apoptotic/necrotic cells. A significant increase in the apoptotic population was observed in Ramos cells treated with FWGP for as little as 1 hour (35.1 ± 3.8%) when compared to untreated controls (12.8 ± 6.4%), and reached a maximum (46.8 ± 10.4%) after 24 hours of treatment (**Fig 2A**). The late apoptotic/necrotic population consistently increased over time, and was significantly higher than untreated controls after 24 and 48 hours of treatment. Similar results were obtained for Raji cells (not shown). Activated caspases were detected in 47.25 ± 17.75% of Ramos cells treated with FWGP for 48 hours, versus 9.24 ± 0.46% of untreated (control) cells (p<0.01, **Fig 2B**). This increase was maintained after 72 hours of incubation (p<0.05). No statistically significant difference was observed at early time points; however, caspase activation was apparent as early as 24 hours (16.15 ± 0.07% vs 9.52 ± 0.06% for treated vs control, respectively). Cell cycle analysis indicated a decrease in the G_0_/G_1_ population with a concomitant increase in the S population (**Fig 2C and S2 Fig**), this became more evident after 24 hours of incubation with FWGP and maintained, albeit to a lesser degree, through 72 hours. There was no significant change in the G_2_/M population. These results suggest that FWGP blocks progression through the S phase of the cell cycle. In agreement with the apoptosis results previously described, there was a marked increase in the subG_1_ population, indicative of dead or dying fragmented cells.

To further examine the direct effects of FWGP on cancer cells at the molecular level, we performed qPCR on treated (2, 6, 12, 48 h) and control Raji cells using an apoptosis and survival pre-designed panel. Consistent with the apoptotic phenotype presented above, treatment with FWGP resulted in early (2h) upregulation of pro-apoptotic genes of the BCL2 family (BAK1, BAD, BAX, BCL10), followed by downregulation of anti-apoptotic AKT1 and upregulation of tumor suppressor TP53 (6h), and upregulation of caspase genes (12-48h, **S3 Fig**). Pro-apoptotic members of the tumor necrosis factor superfamily (TRAIL receptors 1 and 2, TNF) were also upregulated at early time points, as well as the Fas receptor and FADD. In agreement with cell cycle arrest at G1, we observed downregulation of cyclin-dependent kinase CDK1 and upregulation of CDK inhibitors p21, p27 and p16 (**S3 Fig**, see also **S1 Table** for complete qPCR data). To validate some of the qPCR data Immunoblot analysis of selected proteins showed FWGP induced a marked decrease in AKT and increase in BAK, BAD and p53 protein levels by 24–48 h (**Fig 2D**), consistent with earlier changes in message levels.

### *In vivo* lymphomacidal activity of fermented wheat germ extract and fermented wheat germ proteins

**Fig 3:**
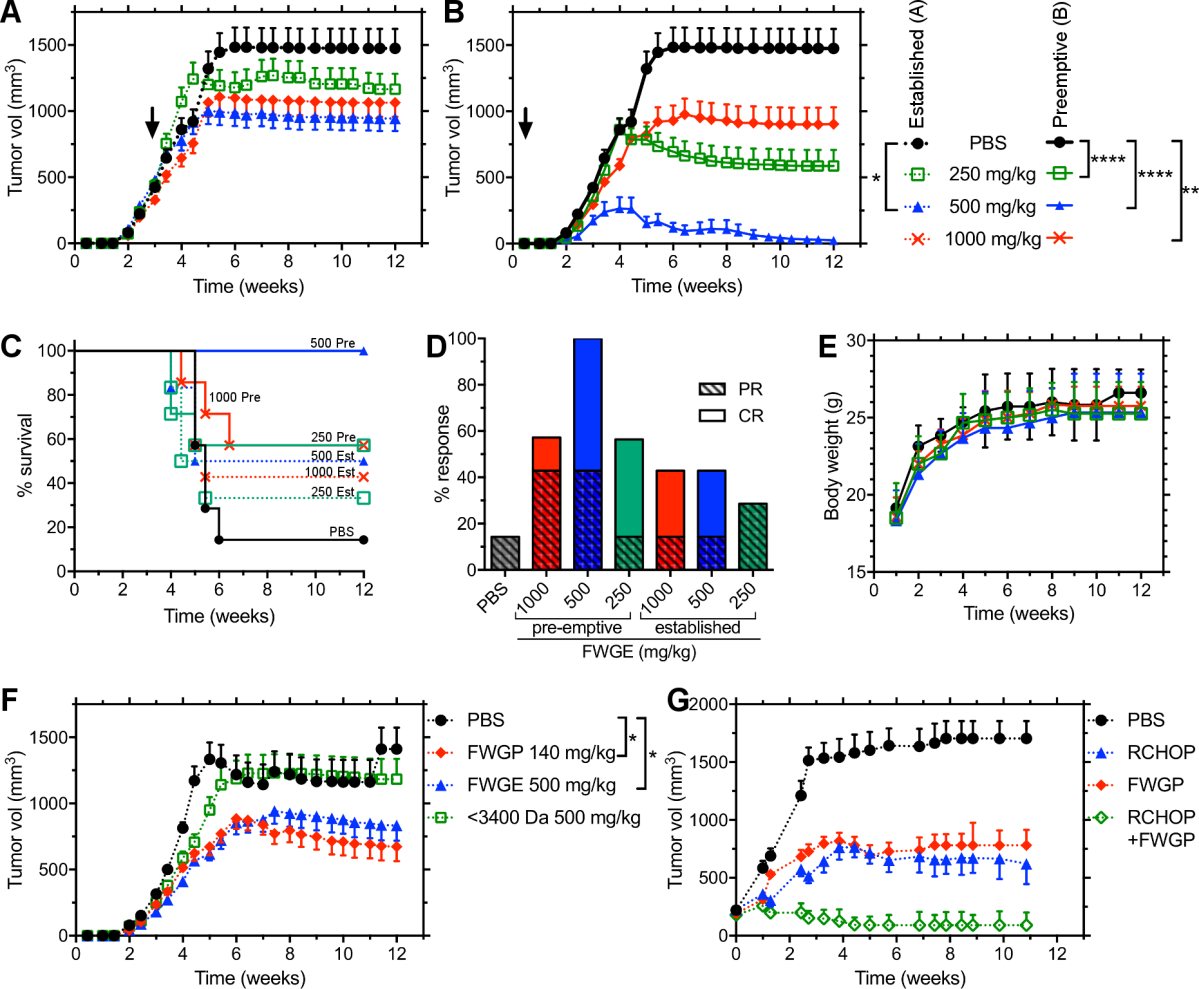
*In vivo* activity of FWGE and FWGP. Raji cells were implanted in nu/nu mice and animals were treated with 250, 500 or 1000 mg/kg of FWGE by gastric gavage 5 days/week; treatment started after tumor reached >100 mm^3^ **(A)** or on the day the xenograft was implanted **(B);** control animals were treated with PBS. **(C)** Overall survival of animals in A and B. Curves are labeled with dose (mg/kg) and preemptive (Pre) or established (Est) tumor model. **(D)** Tumor progression was recorded as complete response (CR) or partial response (PR) to treatment. **(E)** Toxicity was assessed by monitoring animal weight; additional parameters are presented in S6 Fig **(F)** Animals with established Raji tumors were treated with FWGE, the FWGP subfraction or the subfraction <3400 Da in molecular weight (n = 8 animals/groups, *p<0.05). **(G)** Animals with established Raji tumors were treated with FWGP (140 mg/kg), with the RCHOP regimen or both. Data are presented as the average of tumor volume ± SEM (n = 8 animals/group, *p<0.05; **p<0.01; ****p<0.0001, ANOVA with Holm-Sidak multiple comparison). All animal experiments were performed under IACUC guidelines.

The *in vivo* lymphomacidal effects of FWGE were assessed using nude mice bearing Raji xenografts. Mice with established tumors (>100 mm^3^) were treated with FWGE (250, 500 and 1000 mg/kg); after 12 weeks of treatment there was a significant reduction in the tumor volume in the treated groups when compared to untreated controls (average tumor volume ± SEM for increasing doses and control = 1166±324, 944±404,1064±383, and 1475±287 mm^3^ respectively. **Fig 3A**). To examine how the initial tumor volume influenced FWGE efficacy, treatment was initiated on the same day the xenografts were implanted (pre-emptive). This resulted in significantly less growth of the tumor in mice treated at the same doses (250, 500, and 1000mg/kg) compared to untreated controls (587±274, 24±18, 903±381 vs 1475±287 mm^3^ respectively, **Fig 3B**). Interestingly, the intermediate dose (500 mg/kg) was consistently (**S4 Fig**) the most effective in both treatment schemas. Our in-house produced FWGE had similar *in vivo* activity as the commercial product (**S4 Fig**). Survival was 100% at the end of the 12-week study when animals were treated preemptively and 50% when treated after tumors were established, compared to 18% for the untreated control (**Fig 3C**). In preemptive studies, 8/8 animals treated with the 500 mg/kg dose showed at least partial regression compared to 5/8 animals in the higher and lower doses and 1/8 in the control group (**Fig 3D**). No toxicity was observed at any dose, as evidenced by no changes in activity or body weight (**Fig 3E**) as well as normal renal (BUN, creatinine), hepatic (ALT, AST, ALP, Total bilirubin, albumin, serum protein) and hematologic parameters (WBC, platelets, hemoglobin, hematocrit, MCV, **S5 Fig**).

To compare the *in vivo* efficacy of our semi-purified protein extract (FWGP) to FWGE, Raji-bearing mice were treated with FWGP (140 mg/kg) or FWGE (500 mg/kg). Since 1g of FWGE typically yielded ~280 mg of total protein after ethanol precipitation and size exclusion chromatography, the FWGP dose of 140 mg/kg was chosen as equivalent to the most efficacious dose of FWGE. As shown in **Fig 3F**, FWGP was found to have comparable *in vivo* activity at one third the dose (by total protein) of crude FWGE. The tumor volumes at 12 weeks were 673±218 and 833±308 mm^3^ for FWGP and FWGE, respectively, versus 1411±323 mm^3^ for untreated controls. This result not only confirms *in vivo* activity of the protein fraction but also indicates increased potency. Previous reports suggested small molecules such as benzoquinones were responsible for FWGE activity [20]. Our process to produce FWGP from FWGE eliminates small molecules and leaves primarily proteins. When we examined the FWGE fraction that included everything below 3.4 kDa, no efficacy was found in *in vivo* models (**Fig 3F)** supporting the hypothesis that benzoquinones do not play a major role in the efficacy of FWGE or FWGP.

R-CHOP (rituximab + cyclophosphamide + doxorubicin + vincristine + prednisone) is the current standard of care for aggressive NHL. We compared R-CHOP to FWGP as well as the combination of R-CHOP and FWGP and evaluated the combination therapy in mice with established Raji tumors. As shown in **Fig 3G**, 140 mg/kg FWGP was as effective as the R-CHOP regimen (tumor volume at 10 weeks = 782±134 and 665±177 mm^3^, respectively, compared to 1703±150 mm^3^ for controls). Nine of 10 animals treated with a combination of FWGP + R-CHOP showed complete regression, with a tumor volume at the end of the experiment of 91± mm^3^ (representing only 1 animal that did not achieve a CR; 9/10 animals showed complete regression with no palpable tumor).

### FWGP enhances NK cell-mediated tumor eradication

**Fig 4:**
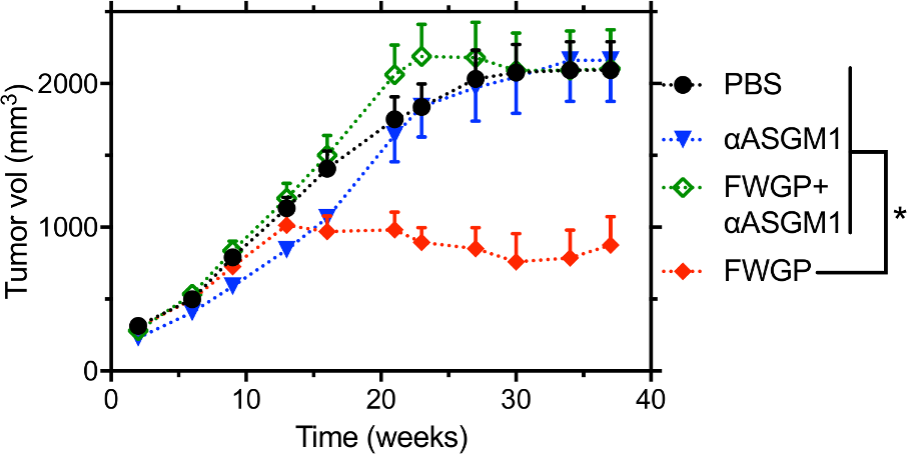
FWGP efficacy depends on NK cell activity *in vivo*. nu/nu mice bearing established Raji tumors were treated with FWGP (140 mg/kg) with or without the NK-cell depleting antibody anti-ASGM1. Controls received either PBS or the antibody only. Data points represent average tumor volume ± SEM (n = 8 animals/group, *p<0.05, ANOVA with Holm-Sidak multiple comparison).

**Fig 5:**
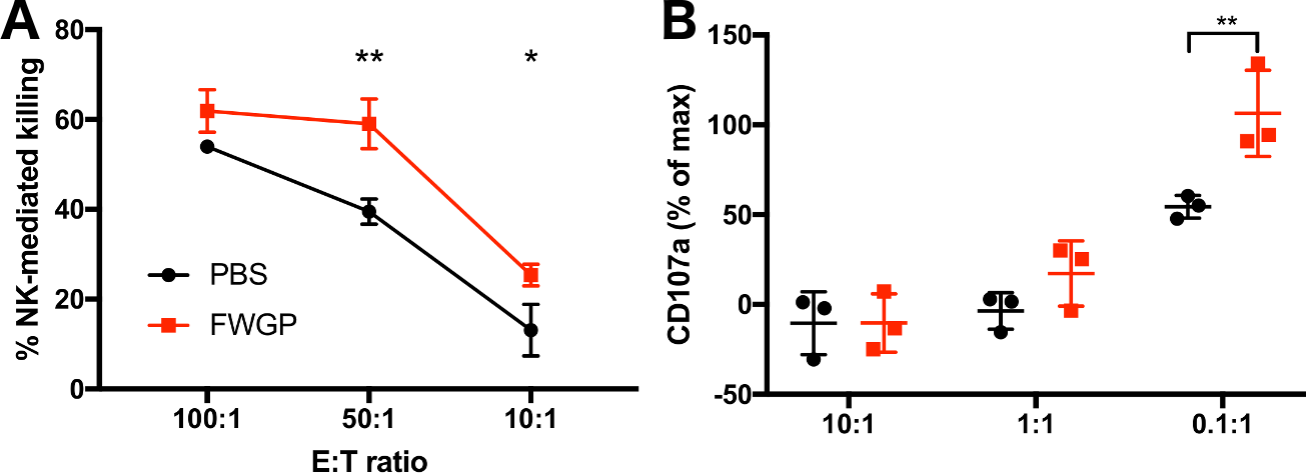
FWGP activates NK cell in immunocompetent mice. BALB/c animals were treated with FWGP (140 mg/kg) or PBS for 3 days before spleens were dissected. Splenocytes were T-cell depleted and used as effector cells in functional assays. **(A)** For killing assays, target cells were CFSE-labeled YAC-1. Data points represent the mean±SD of the % CFSE^+^FVD^+^ cells. **(B)** For degranulation assays YAC-1 cells were used as target. Data points represent the median±SD fluorescence intensity (as % of max) of CD107a staining. Maximum degranulation was defined as the CD107a signal intensity in cells stimulated with PMA+ionomycin (n = 3; *p<0.05, **p<0.01; 2-way ANOVA with Sidak’s multiple comparison test).

Although rigorous studies are lacking, FWGE has been reported to have immunomodulatory properties, including stimulatory effects on mouse lymphocytes *in vitro* [11], immune-restoring effects in thymectomized animals [9] and decreased expression of MHC-I on the tumor cell surface [13]. Since we demonstrated efficacy in the nu/nu xenograft model and this model lacks T cells, but retains natural killer (NK) cell numbers and activity, we hypothesized that FWGP could make cancer cells more susceptible to NK cell surveillance. To test the hypothesis that the observed *in vivo* efficacy of FWGP is, at least in part, due to increased NK anti-tumor activity, we performed xenograft experiments combining FWGP treatment and NK cell depletion. Consistent with previous results, FWGP treatment resulted in significant tumor reduction (tumor volume at 5 weeks = 877±280 versus 2093±395 mm^3^ for FWGP and PBS, respectively). However, animals treated with FWGP and concomitantly depleted NK cells had tumor volumes of 2104±541 mm^3^ with the tumor volume being no different from PBS-treated controls. (**Fig 4**). Animals treated with the depleting antibody (anti-ASGM1) had no effect (tumor volume = 2161±571 mm^3^). NK cell depletion was confirmed by flow cytometry (**S6 Fig**). To investigate the effects of FWGP on the intact immune system, we treated immunocompetent BALB/c mice with FWGP for 3 days and examined PBMC subsets isolated from the spleen on day 4. Immune cell subpopulations (T cells, B cells, granulocytes, monocytes) in treated animals were not significantly different from controls, except for a modest increase in NK cell numbers (10.5±0.8% vs 7.4±0.3%, *p* = 0.0039, **S7 Fig**). However, we observed a significant increase in NK-cell killing activity, as assayed by flow cytometry of T-cell depleted splenocytes (effector) and CFSE-labeled YAC-1 (target) cells (**Fig 5A**). NK cells from treated animals killed 59.1±5.5 and 25.4±2.4% of target cells at E:T ratios of 50:1 and 10:1 respectively, while cells from control animals killed 39.5±2.8 and 13.1±5.7% (*p* = 0.0020 and 0.0324, respectively for 50:1 and 10:1 ratios). This result is further supported by increased degranulation of NK cells from treated vs control animals (106.5±24.0 vs 54.4±6.4% at E:T = 0.1:1), measured by CD107a staining intensity in the CD3^-^CD49b^+^ subpopulation of T-cell depleted splenocytes (**Fig 5B**).

### FWGP stimulates human NK cells

**Fig 6:**
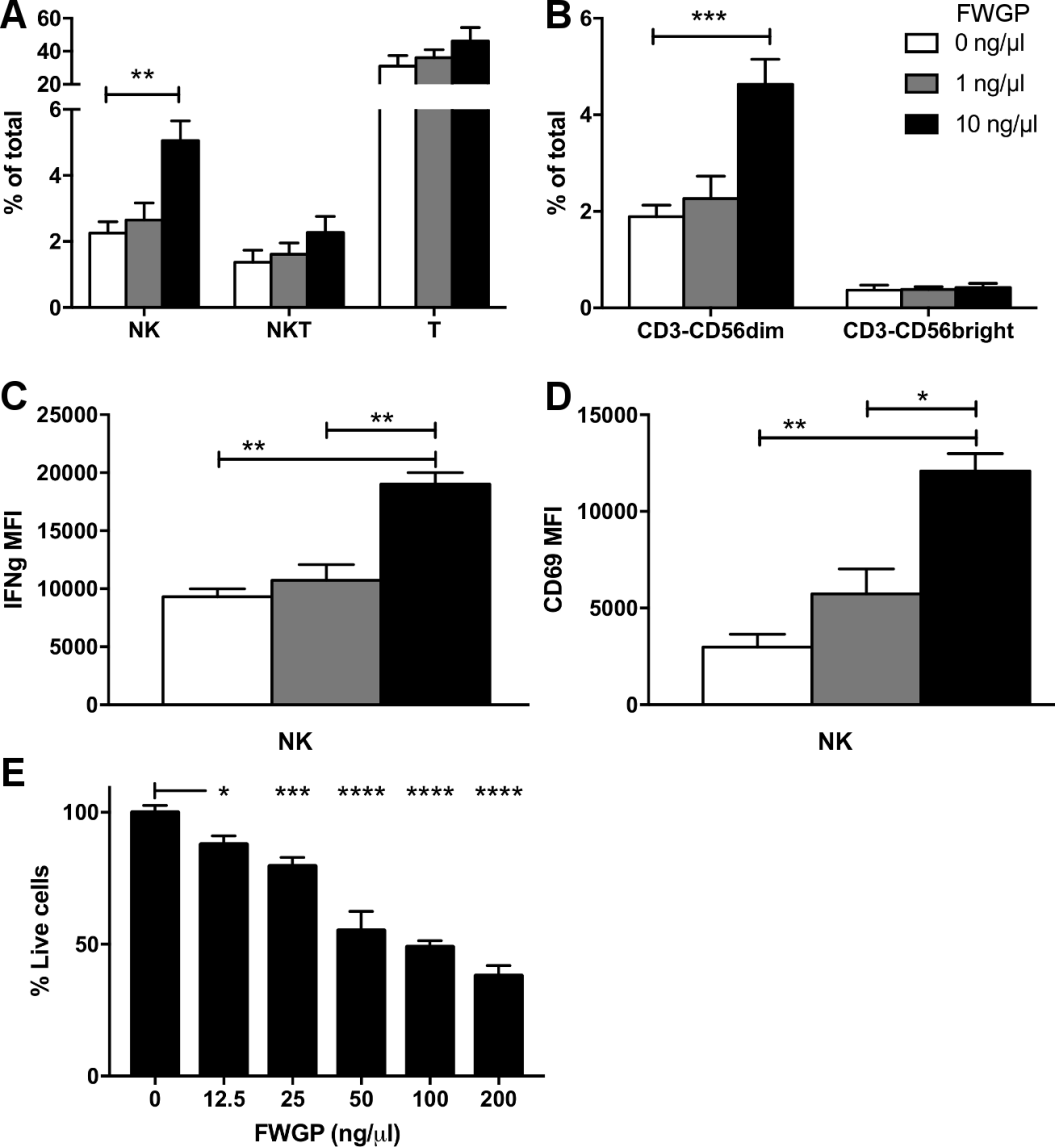
FWGP activates human NK cells *ex vivo*. PBMCs from healthy donors were incubated with FWGP (at the indicated concentrations) or medium only (control) for 16 h. **(A)** NK (CD3^-^CD56^+^), NKT (CD3^+^CD56^+^) and T (CD3^+^CD56^-^) populations as % of total live cells. **(B)** CD56^dim^ and CD56^bright^ subpopulations as % of total live cells (n = 3; **p<0.01, ***p<0.001; 2-way ANOVA with Tukey’s multiple comparison test). NK cell activation was assessed as median fluorescence intensity of IFNγ **(C)** and CD69 **(D)** in the CD3^-^CD56^+^ population (n = 3; *p<0.05, **p<0.01; ANOVA with Dunnet’s multiple comparison test). **(E)** Killing activity against Ramos cells at E:T = 1:1. Bars indicate the mean±SD of % live cells after 24 h contact time, relative to Ramos cells incubated with untreated PBMCs controls (n = 3; *p<0.05, ***p<0.001, ****p<0.0001; ANOVA with Dunnet’s multiple comparison test).

To test if the results obtained in mice can be extrapolated to human NK cells, we performed an *ex vivo* experiment by incubating PBMCs from healthy donors with 1 or 10 ng/μl FWGP overnight. In agreement with the results from mouse experiments, when compared to untreated controls, incubation of human PBMCs with FWGP resulted in an increase in the CD3^-^CD56^+^ population (2.2±0.4 vs 2.6±0.5 vs 5.1±0.6 for 0, 1 and 10 ng/μl, respectively; *p*<0.01), with no changes in the CD3^+^CD56^-/+^ populations. The increase in the NK cell compartment was driven by an increase in the CD56^dim^ subset, with no changes in CD56^bright^ cells (**Fig 6A** and **6B**). Importantly, FWGP caused increased production of IFNγ in human NK cells (MFI = 9305±694 vs 10733±1358 vs 19000±1010, *p*<0.01), and increased surface levels of the early activation marker CD69 (MFI = 2982±669 vs 5738±1283 vs 12091±899, *p*<0.01 and *p*<0.05, **Fig 6C** and **6D**). Finally, to examine the effect of FWGP on killing activity, human PBMCs were incubated with increasing concentrations of FWGP overnight, washed and incubated with target cells (Ramos) for 24 hours. There was a dose-dependent increase in killing activity of PBMCs (**Fig 6E**). The lowest dose tested (12.5 ng/μl) resulted in 87.88±3.22% viable cells, a small but significant decrease when compared to controls (99.97±2.6%, *p*<0.05). At the highest dose tested, only 38.0±3.8% (*p*<0.0001) target cells remained alive, representing a 2.6-fold increase in killing activity. To ensure that the effects of FWGP on PBMC numbers did not confound the interpretation, PBMC cell numbers were examined after incubation with the indicated dose of FWGP. As seen with B lymphocytes (see Fig 1A), the effect of FWGP on PBMC numbers was modest, however to ensure that this did not confound interpretation of the cytotoxicity assay, PBMC numbers were adjusted and normalized prior to each experiment.

## Discussion

Cancer patients are more frequently turning to complementary medicine and nutraceuticals, especially when standard treatment options fail. FWGE is a nutraceutical that has been reported to possess unique “cancer-fighting” characteristics [28]. Since its first description in the late 1990s [9–11], anti-cancer activity has been reported for a variety of human tumors [15, 17, 19, 20, 22–24, 37–39]. Most of these studies evaluate cytotoxic activity on cancer-derived cell lines either *in vitro* or in xenograft models. A few studies report FWGE has immunomodulatory properties [11, 13, 27–29, 40], however these studies lack a rigorous analysis. Here we provide in-depth examination of the cytotoxic effects of FWGE on lymphoma cells *in vitro* and *in vivo*, explore the mechanisms of action, and present evidence that it enhances NK-cell mediated tumor eradication. We further found that these activities are present in a protein subfraction (FWGP), contradicting the current paradigm that benzoquinones are responsible for FWGE anti-cancer properties.

FWGE produced in-house by fermenting wheat germ with *S*. *cerevisiae* had equivalent lymphomacidal activity to commercially available AVEMAR^®^ and demonstrated significant *in vitro* activity in a panel of 9 NHL cell lines that represent the majority of clinically-relevant subtypes of NHL. The LD_50_ nearly doubled for primary human B cells, suggesting FWGE has greater activity in malignant versus nonmalignant B cells. While there have been, to our knowledge, no attempts to purify and identify the active components of FWGE, a benzoquinone (DMBQ) has been suggested to be the active constituent [9, 10, 27, 39] and is used to quantify and standardize the activity of AVEMAR^®^. However, this has not been proven beyond an observational correlation, and indeed early studies indicated that DMBQ alone cannot be responsible for the immunostimulatory properties of FWGE [11]. Our results suggest that peptide components of FWGE are responsible for the anti-cancer activities we report here, since such activities: i) are present in an ethanol-insoluble extract, further purified to 10–100 kDa molecular weight components; ii) the activity is lost upon treatment with proteinase K, and iii) the active component(s) are heat-sensitive. WGA is known to be cytotoxic [36]; however, WGA-depleted FWGP remained highly effective, demonstrating that the lymphomacidal effects of FWGP are not mediated by this agglutinin. Previous studies have suggested that FWGE mediates cell killing, in part, by directly inducing apoptosis [12, 41]. We confirmed apoptosis-inducing activity of FWGP by increased Annexin-V staining and increased caspase activity of NHL cells that had been treated with FWGP. FWGE has been reported to induce cell cycle arrest [39] by blocking progression through the G1 phase [14], and possibly by downregulating cyclin D1 [22]. Our results, however, suggest that FWGP blocks successful completion of the S phase, as the treatment of NHL cells with FWGP resulted in a decrease in the G1 population with a concomitant increase in the S-phase population. It may be argued that FWGE components absent in FWGP may be responsible for the G1 blockade previously reported. Regardless, our results of *in vitro* cytotoxicity, apoptosis and cell cycle analysis confirm that the cytostatic/cytolytic properties previously reported for FWGE are present in a protein subfraction, FWGP.

FWGE and FWGP reproducibly demonstrated effective *in vivo* lymphomacidal activity at several doses. Importantly, our semi-purified fraction FWGP showed higher potency. While there was a clear and reproducible dose-response effect, it was interesting that the intermediate dose was the most effective, producing a greater than 10-fold reduction in tumor volume after 24 weeks of therapy when compared to the control; when compared to the lowest dose there was a 5-fold reduction in tumor volume. Furthermore, the FWGE purification fraction that contained small molecules (<3400 Da) had no significant efficacy, again suggesting that small molecules such as DMBQ are not the active components for the response seen *in vivo*.

Many immune-based therapeutics are more effective with lower tumor burdens [42] thus xenograft studies using FWGE were repeated using a preemptive approach; this demonstrated even greater efficacy. However, higher doses were consistently inferior in efficacy. FWGE has a wide therapeutic window, however, cytotoxic effects are indeed seen at very high doses in normal lymphocytes *in vitro* (this study and [12]). While PD/PK measurement were beyond the purpose of this study, it is possible that higher oral doses of FWGP result in blood levels high enough to off-balance anti-tumor activity with immune cell toxicity. Although merely hypothetical, this possibility remains appealing in view of our results that indicate that NK-cell mediated tumor eradication is a strong component of FWGP mechanism of action *in vivo*. This has important implications when considering that this agent in the form of AVEMAR^®^, is available to the public and no dose-finding studies have been done.

These results support the hypothesis that FWGP enhances innate anti-tumor immunity. FWGE has been reported to increase blastic transformation of peripheral blood T cells by concanavalin A [11], to reduce graft survival in a coisogenic skin transplantation model [11] and to reduce production of IL-4 and IL-10 in a systemic lupus erythematosus model [27], supporting its immunomodulatory properties.

Of particular interest to this work, FWGE has been reported to induce downregulation of MHC-I proteins in tumor T and B cell lines [13], leading to the hypothesis that this would make tumor cells more “visible” to NK cells and hence improve immune tumor eradication. While this may indeed be true, our results further suggest that FWGP activates NK cells *per se*, as we observed an increase in the degranulation response and increased NK-mediated killing activity in tumor-free, immunocompetent BALB/c mice treated with FWGP. Although initially thought to recognize and eliminate their targets with fast kinetics and no prior sensitization, it is now recognized that NK cells attain full effector function only after they have been licensed by engaging self MHC-I [43]. In addition, NK cells need to be primed, for example, by trans-presentation of interleukin 15 [44], and interleukin 18 has been reported to regulate NK cell IFNγ production [45]. “Conditioning” through constant triggering of Toll-like receptor 3 has been proposed to ensure immediate potent NK cell response to cytokine stimulation [46], although the ligands required for such conditioning are unknown. Whatever the mechanisms are for NK cell hyporesponsiveness to tumors, it is tempting to hypothesize that components of FWGP promote a more responsive state of NK cells. Further studies focusing on the ability of FWGP components to trigger/block NK cells stimulatory and/or inhibitory receptors may answer this question. Finally, our *in vivo* studies used oral administration of FWGP. The gut microbiota influences both local and systemic immune function [47, 48], and has been shown to influence cancer response to immunotherapy [49]. Therefore, the effects of FWGP on gut microbiota and immunity warrants further investigation.

## Conclusions

While novel targeted chemotherapy and immune-therapeutic approaches have revolutionized the way lymphoma is treated, many patients will eventually succumb to it. The toxicity of many of the currently available drugs limits their efficacy, particularly in the elderly, whom lymphoma most commonly afflicts. Here we present evidence that a protein fraction from fermented wheat germ has direct lymphomacidal activity *in vitro*. This activity is dependent on protein components and not DMBQ as previously reported, since protease or heat treatment resulted in loss of activity. Importantly, a protein extract from fermented wheat germ has in vivo lymphomacidal activity, yet it has no appreciable toxicity even at the highest doses tested. Remarkably, treatment with FWGP alone was as effective as the R-CHOP regimen which is the standard of care for many patients with lymphoma. This activity was dependent on NK cells, as efficacy was lost upon NK cell depletion. Furthermore, treatment of tumor-free, immunocompetent animals resulted in increased NK cell killing activity, increased degranulation and increased IFNγ production upon *ex vivo* stimulation. Translation of this product into allopathic medicine could constitute a novel non-toxic alternative for NHL patients. Furthermore, its use in conjunction with the current standard of care could allow for lower doses of chemotherapy, thereby overcoming toxicity limitations which would have a significant impact in patients’ outcome and quality of life. Clinical studies should assess the efficacy of the current formulation of this promising therapeutic. It is clear that it will be necessary to identify the active compound(s) of FWGP. Studies are currently ongoing in this regard.

## Supporting information

S1 Fig *In vitro* FWGP cytotoxic activity is not due to WGA. Raji cells were incubated with increasing concentrations of FWGP that had previously been depleted of WGA by immunoprecipitation. Bars indicate mean±SD live cells relative to untreated controls after 72 hours.
(TIF)

S2 Fig Cell cycle. Raji cells were incubated with FWGP (200 μg/ml) or medium only (control) for the indicated times, fixed/permeabilized and stained with propidium iodide. Plots represent flow cytometry data with populations calculated by FlowJo’s unidimensional algorithm.
(TIF)

S3 Fig FWGP effects on apoptosis and survival pathways. Quantitative real-time PCR assays were performed using the H384 panel (Bio-Rad PrimePCR) to examine over 350 genes associated with cell survival and apoptosis. Total RNA was extracted from control and treated Raji cells at the indicated time points. Color code in the clustergram indicates standardized gene expression (red = high, green = low). Data were analyzed with Bio-Rad’s PrimePCR software. Only genes mentioned in the main article are shown. The complete data are available as S1 Table.
(TIF)

S4 Fig FWGE has lymphomacidal activity in a murine model of human NHL. Data from 3 independent experiments in which nu/nu mice bearing Raji NHL xenogratfs were treated with 3 different batches of fermented wheat germ extract prepared in our laboratory (FWGE), the commercially available product Avemar™ (Ave) or PBS as a control. Colors indicate different doses (n = 10 animals/experiment/group).
(TIF)

S5 Fig Toxicity. No toxicity was observed during treatment with either FWGE or FWGP, as assesses by blood **(A, B, C)**, liver **(D, E)** and renal **(F)** function (n = 10 animals/group). WBC: white blood cells; RBC: red blood cells; Hb: hemoglobin; Ht: hematocrit; MCV: mean corpuscular volume; MCH: mean corpuscular hemoglobin; MCHC: mean corpuscular hemoglobin concentration; N: neutrophils; B: basophils; E: eosinophils; L: lymphocytes; M: monocytes; ALT: alanine aminotransferase; AST: aspartate aminotransferase; ALP: alkaline phosphatase; TSB: total serum bilirubin; Alb: serum albumin; Prot: total serum protein; BUN: blood urea nitrogen; Cr: creatininemia.
(TIF)

S6 Fig NK-cell depletion. Splenocytes from PBS control (A) and NK-depleted (B) animals (1 each) were stained with with anti-CD49b. Plots represent flow cytometry data with gating strategy.
(TIF)

S7 Fig Immune phenotypic profiling. Splenocytes from BALB/c mice treated with FWGP (140 μg/ml) or PBS (control) for 3 days were stained for flow cytometry. Immune populations were defined as follows: B cells, CD45+CD11b-CD19+; T cells, CD45+CD11b-CD3+; Myeloid cells, CD45+CD11b+; Tc, CD45+CD11b-CD3+CD4-CD8+; Th, CD45+CD11b-CD3+CD4+CD8-; NK cells, CD45+CD11b-CD19-CD3-CD49b+; NKT cells, CD45+CD11b-CD3+CD49b+. Data were gated for single cells and live cells before gating for lineage markers. Bars represent mean±SD.
(TIF)

S1 Table Cell survival and apoptosis panel. Quantitative PCR data from control and treated Raji cells at the indicated time points. Data were analyzed with Bio-Rad’s PrimePCR software.
(CSV)
