# Supplementary figures and images for: A purified, fermented, extract of Triticum aestivum has lymphomacidal activity mediated via natural killer cell activation
Source: PLoS One. 2018 Jan 5;13(1):e0190860. doi: 10.1371/journal.pone.0190860 (PMC5755921; doi:10.1371/journal.pone.0190860)

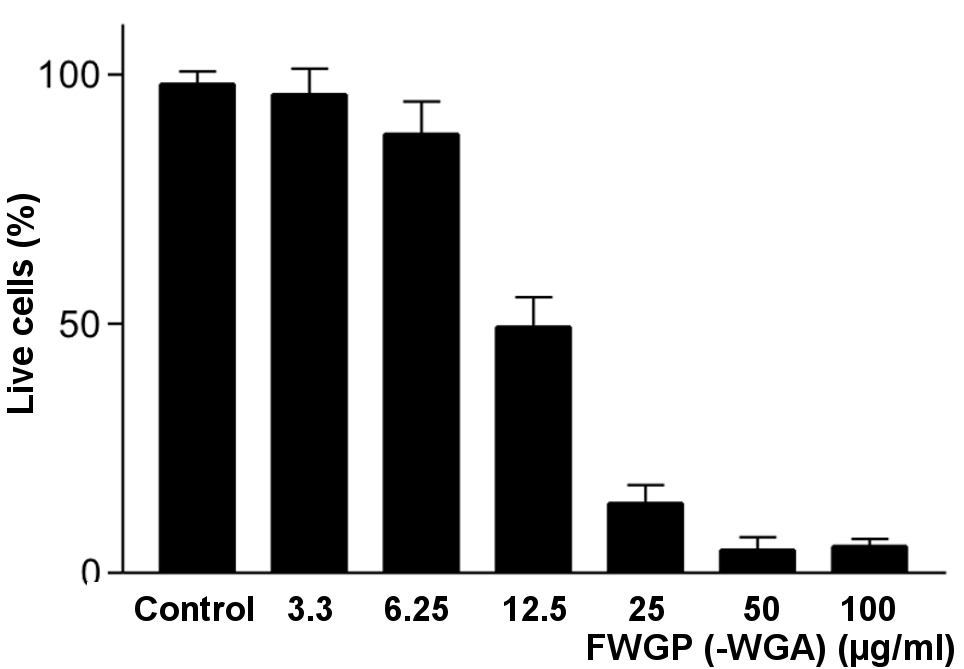

Supplement: S1 Fig — Raji cells were incubated with increasing concentrations of FWGP that had previously been depleted of WGA by immunoprecipitation. Bars indicate mean±SD live cells relative to untreated controls after 72 hours. (TIF) [file pone.0190860.s001.tif]

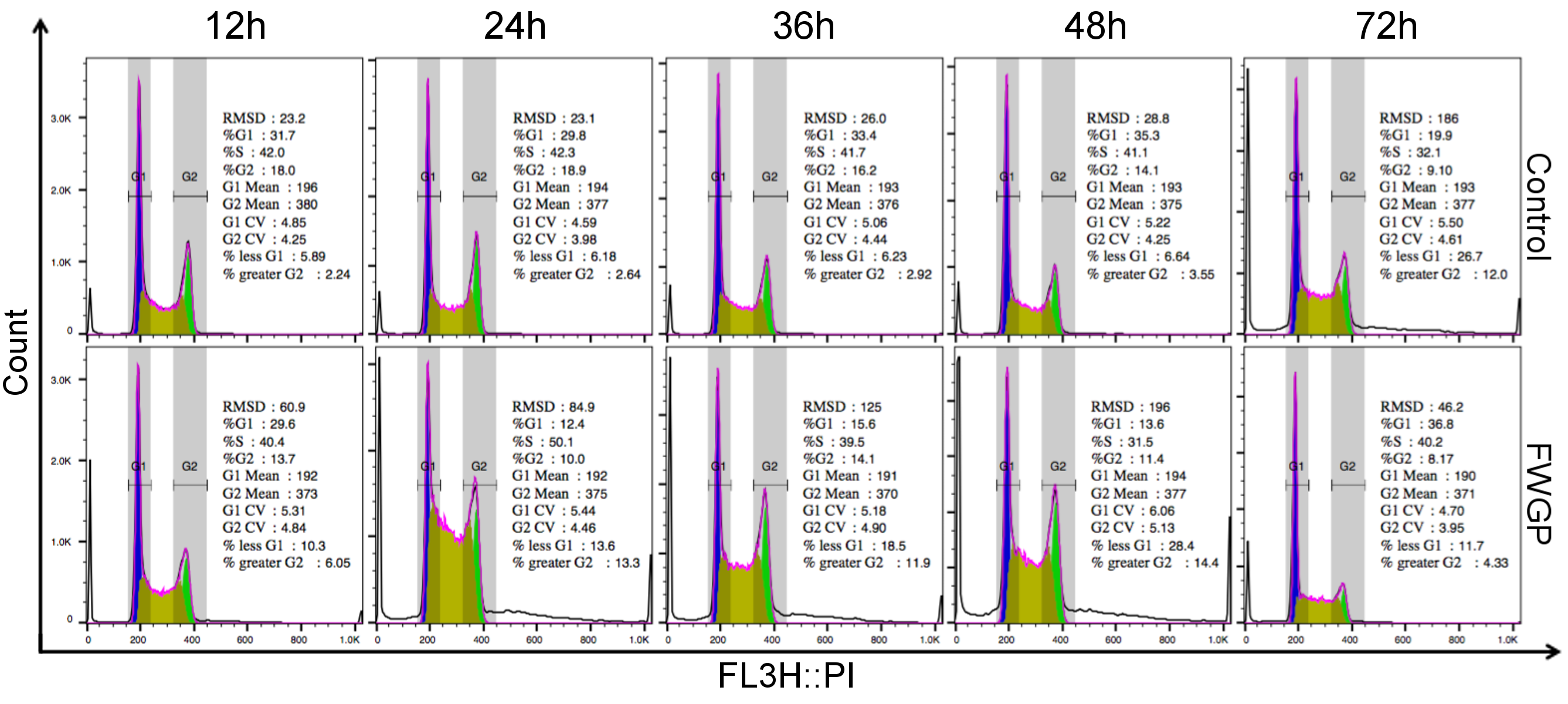

Supplement: S2 Fig — Raji cells were incubated with FWGP (200 μg/ml) or medium only (control) for the indicated times, fixed/permeabilized and stained with propidium iodide. Plots represent flow cytometry data with populations calculated by FlowJo’s unidimensional algorithm. (TIF) [file pone.0190860.s002.tif]

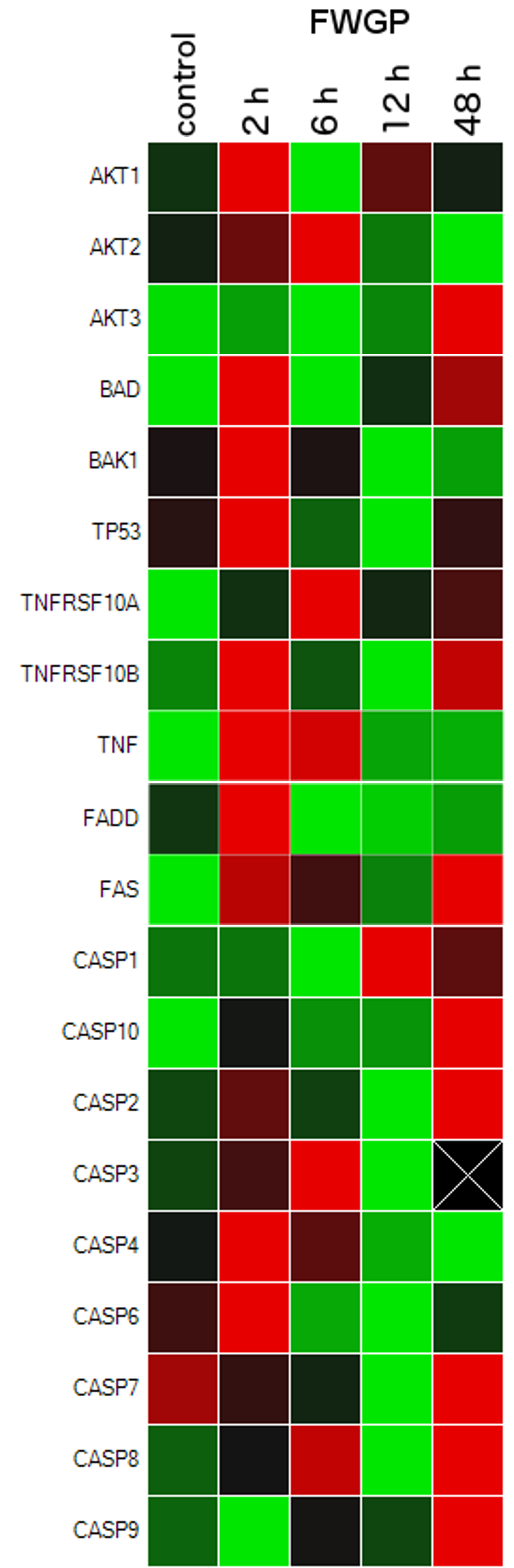

Supplement: S3 Fig — Quantitative real-time PCR assays were performed using the H384 panel (Bio-Rad PrimePCR) to examine over 350 genes associated with cell survival and apoptosis. Total RNA was extracted from control and treated Raji cells at the indicated time points. Color code in the clustergram indicates standardized gene expression (red = high, green = low). Data were analyzed with Bio-Rad’s PrimePCR software. Only genes mentioned in the main article are shown. The complete data are available as S1 Table. (TIF) [file pone.0190860.s003.tif]

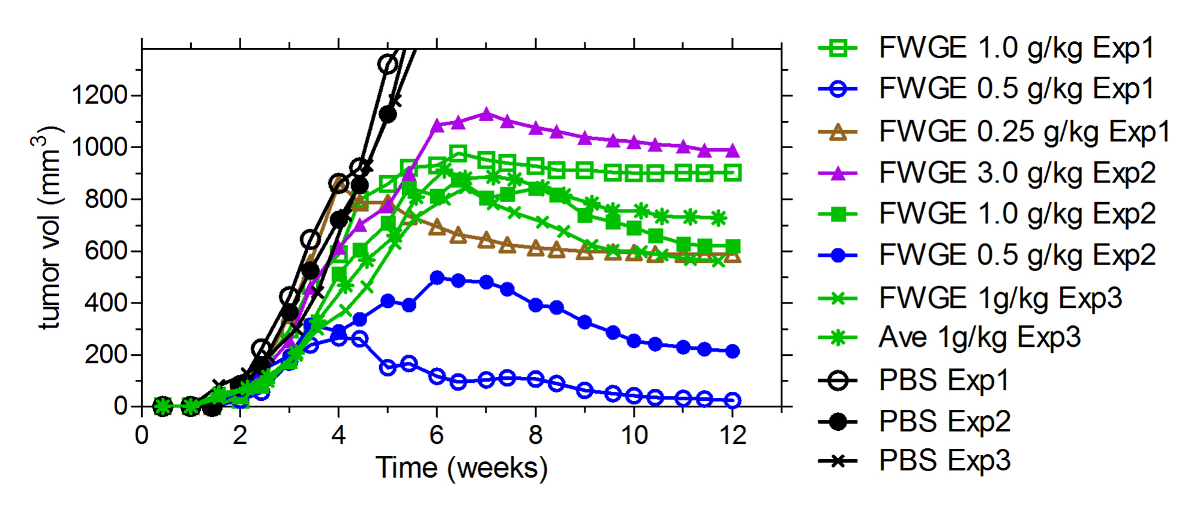

Supplement: S4 Fig — Data from 3 independent experiments in which nu/nu mice bearing Raji NHL xenogratfs were treated with 3 different batches of fermented wheat germ extract prepared in our laboratory (FWGE), the commercially available product Avemar™ (Ave) or PBS as a control. Colors indicate different doses (n = 10 animals/experiment/group). (TIF) [file pone.0190860.s004.tif]

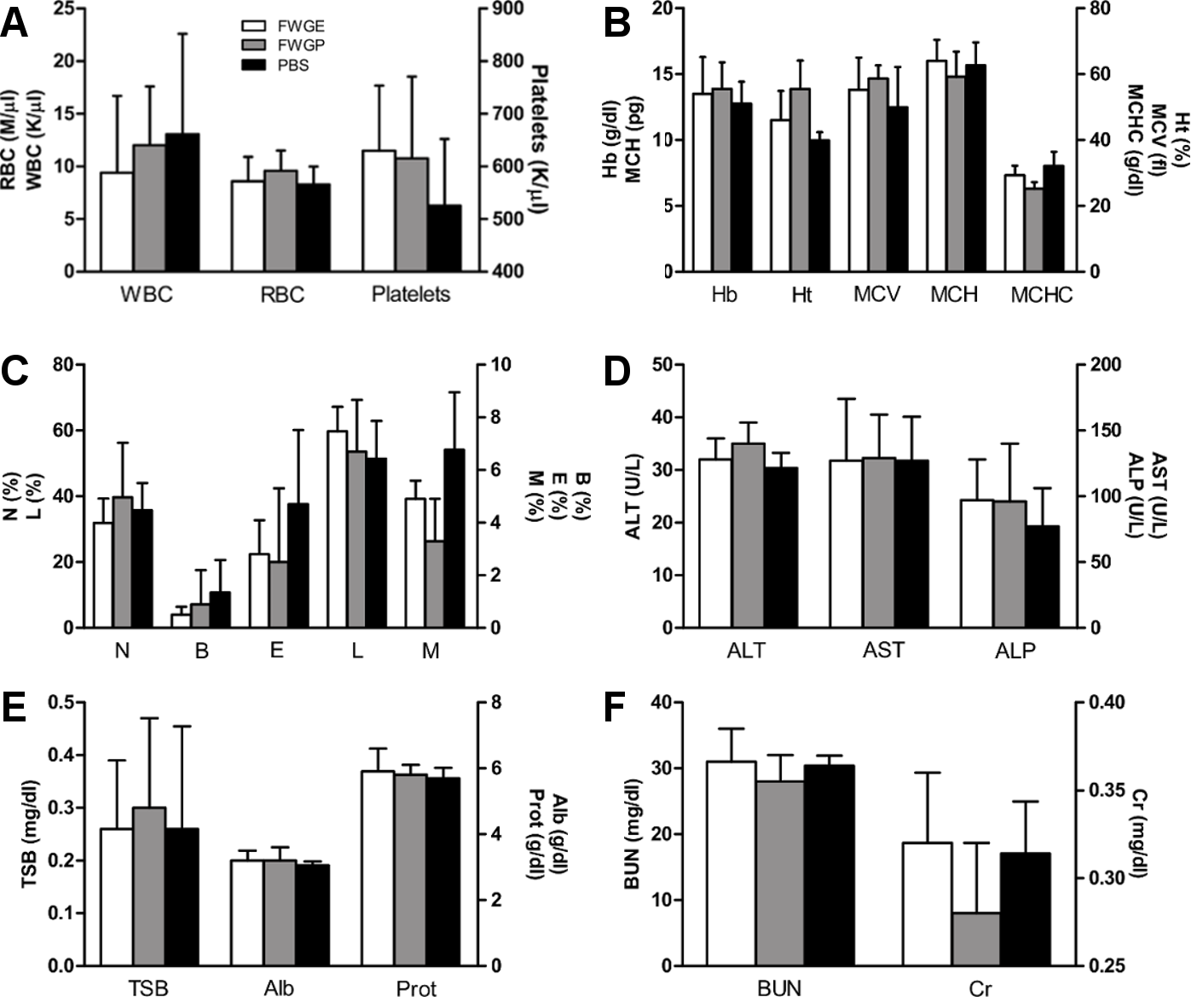

Supplement: S5 Fig — No toxicity was observed during treatment with either FWGE or FWGP, as assesses by blood (A, B, C), liver (D, E) and renal (F) function (n = 10 animals/group). WBC: white blood cells; RBC: red blood cells; Hb: hemoglobin; Ht: hematocrit; MCV: mean corpuscular volume; MCH: mean corpuscular hemoglobin; MCHC: mean corpuscular hemoglobin concentration; N: neutrophils; B: basophils; E: eosinophils; L: lymphocytes; M: monocytes; ALT: alanine aminotransferase; AST: aspartate aminotransferase; ALP: alkaline phosphatase; TSB: total serum bilirubin; Alb: serum albumin; Prot: total serum protein; BUN: blood urea nitrogen; Cr: creatininemia. (TIF) [file pone.0190860.s005.tif]

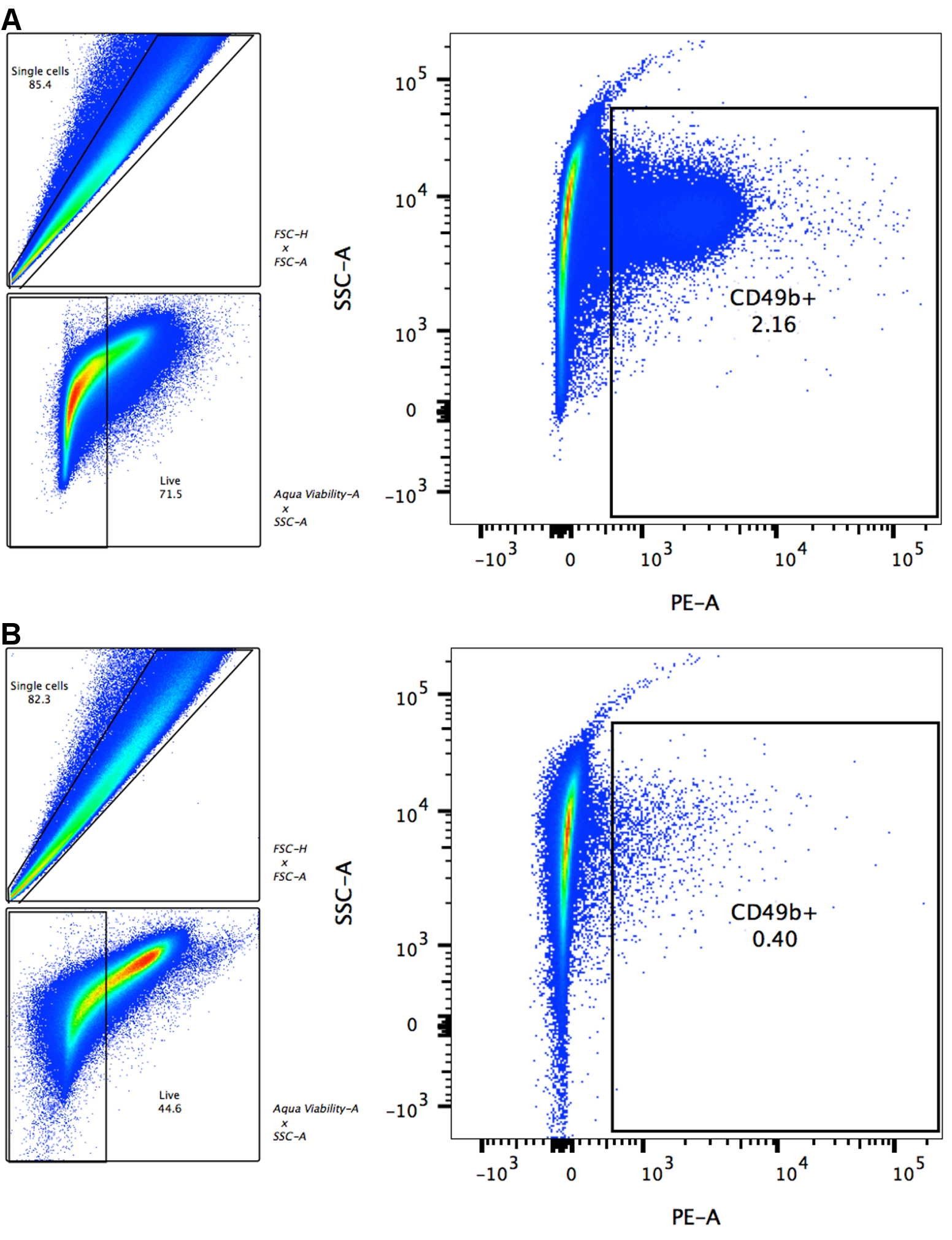

Supplement: S6 Fig — Splenocytes from PBS control (A) and NK-depleted (B) animals (1 each) were stained with with anti-CD49b. Plots represent flow cytometry data with gating strategy. (TIF) [file pone.0190860.s006.tif]

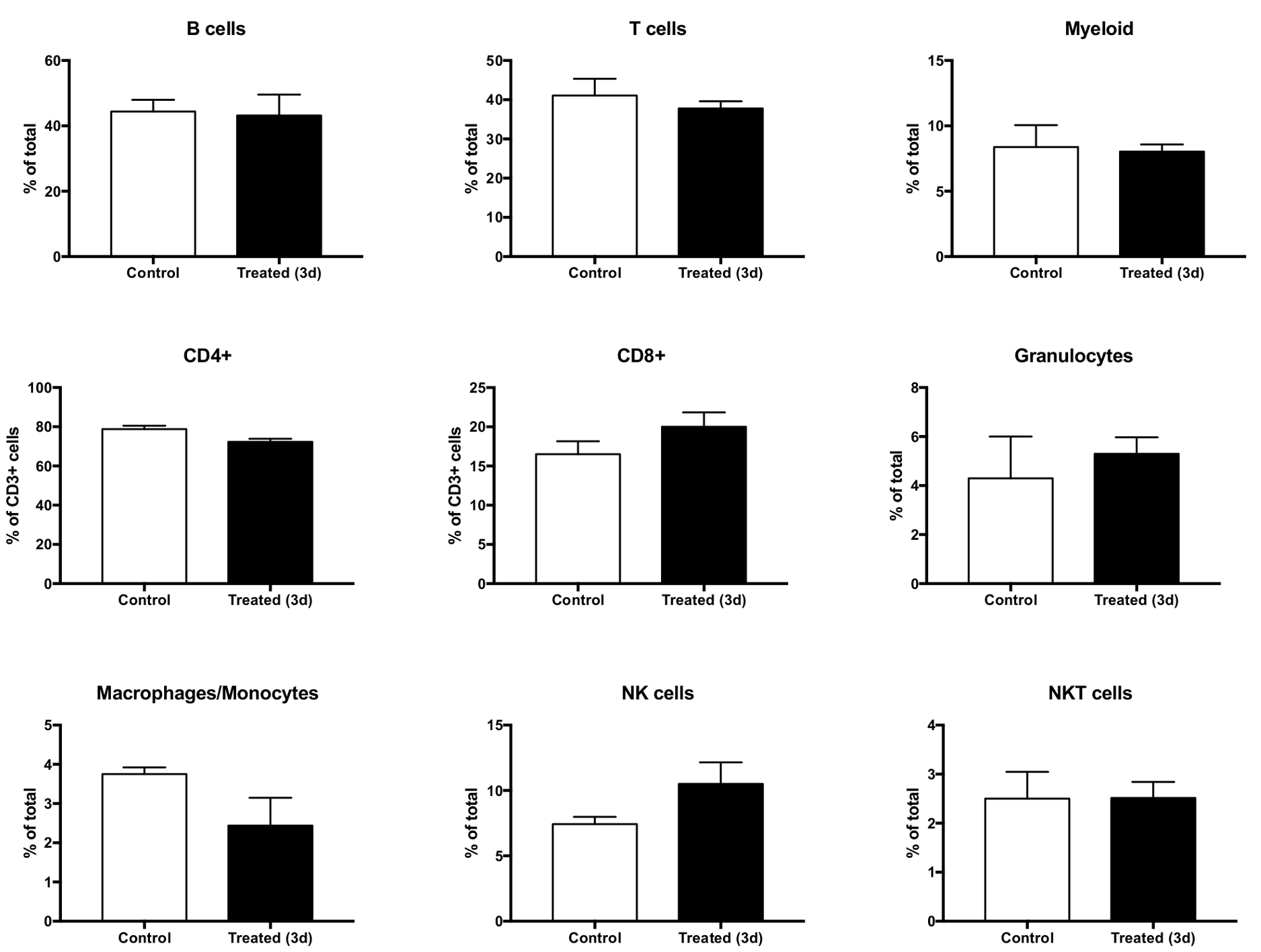

Supplement: S7 Fig — Splenocytes from BALB/c mice treated with FWGP (140 μg/ml) or PBS (control) for 3 days were stained for flow cytometry. Immune populations were defined as follows: B cells, CD45+CD11b-CD19+; T cells, CD45+CD11b-CD3+; Myeloid cells, CD45+CD11b+; Tc, CD45+CD11b-CD3+CD4-CD8+; Th, CD45+CD11b-CD3+CD4+CD8-; NK cells, CD45+CD11b-CD19-CD3-CD49b+; NKT cells, CD45+CD11b-CD3+CD49b+. Data were gated for single cells and live cells before gating for lineage markers. Bars represent mean±SD. (TIF) [file pone.0190860.s007.tif]
